# In vitro study examines posterior torque impact on 3D mechanics of anterior teeth in clear aligner treatment

**DOI:** 10.1186/s12903-024-04240-7

**Published:** 2024-04-23

**Authors:** Yongjie Fan, Xin Zhang

**Affiliations:** Department of Orthodontics, The Fourth Affiliated Hospital of Inner Mongolia Medical University, Baotou, 014030 China

**Keywords:** Clear aligners, Extraction, Torque, Three-dimensional forces

## Abstract

**Introduction:**

This study utilizes investigate the impact of posterior torques on the three-dimensional force exerted on the lower anterior teeth during the retraction in orthodontic clear aligners treatment.

**Methods:**

Four groups of mandibular dental arch light-cured resin models will be created, including: mandibular posterior teeth with standard torque, mandibular posterior teeth with labial torque, and mandibular posterior teeth with lingual torque. Each group will consist of 12 sets of clear aligners. The aligners will be worn, and measurements will be taken using the six-axis measurement platform to evaluate the three-dimensional force exerted on the lower anterior teeth under various initial torques applied to the mandibular posterior teeth. SPSS 26.0 used for ANOVA analysis, α = 0.05 significance level.

**Results:**

Comparing mandibular posterior teeth with standard torque to those with labial torque, no statistically significant changes were observed in buccolingual force. In the mesiodistal direction, mandibular incisors exhibited a significant decrease in distal force, while canines showed a significant increase. Both findings had a significance level of *P* < 0.05; Lingual torque on mandibular posterior teeth, compared to standard torque, led to a significant increase in lingual force for incisors and a significant increase in labial force for canines in the buccolingual direction (*P* < 0.05). Additionally, mandibular incisors exhibited a significant decrease in distal force in the mesiodistal direction (*P* < 0.05).

**Conclusion:**

Varying initial torques on mandibular posterior teeth significantly impact force on lower anterior teeth. Labial torque reduces lingual force on incisors and increases distal force on canines. Lingual torque increases lingual force on incisors and labial force on canines.

**Supplementary Information:**

The online version contains supplementary material available at 10.1186/s12903-024-04240-7.

## Introduction

Fixed orthodontic treatment is currently the most commonly used method for correcting malocclusion in clinical practice. Its corrective force is transmitted through the deformation of the archwire, from the brackets on the buccal or lingual surface to the orthodontic teeth. Due to the presence of a gap angle between the archwire and the brackets, and the force application point at the resistance center of the teeth on the buccal or lingual side, fixed orthodontic appliances often result in suboptimal control of the teeth [1]. In contrast, removable clear aligners, due to their full coverage of the tooth surface, result in more uniform force distribution across all tooth surfaces, closer to the resistance center of the teeth [2].

Compared with fixed braces, clear aligners have gained increasing popularity among patients and orthodontic professionals due to their aesthetic appeal and comfort advantages [3–6]. With the advancement of digital technology and in-depth research on the biomechanical properties of materials, the scope of treatment with clear aligners has expanded from non-extraction cases to include extraction cases [7–10].

Nevertheless, several studies [11–13] have highlighted the impact of material hardness on clear aligners. They have found that these aligners often fail to maintain their original shape when closing gaps, leading to mesiodistal tilting of molars and the loss of buccolingual torque [14, 15]. As molars play a crucial role as anchor teeth, their positioning during the retraction process significantly influences the force exerted on the anterior teeth. Previous research [16] has demonstrated that different initial axial inclinations of the posterior teeth have a substantial effect on the three-dimensional force on the anterior teeth. However, the specific effects of different torques applied to the posterior teeth on the force exerted on the anterior teeth remain unclear.

In recent years, three-dimensional finite element analysis, pressure sensor testing, and six-axis force sensor testing have become common methods for studying the mechanics of orthodontics. In this experiment, the six-axis force sensor testing method was chosen as the main research method. The reason is that it can comprehensively measure the instantaneous forces in three dimensions on each tooth of the entire lower dental arch and consider the interactions between teeth [17].

Taking these factors into account, the objective of this study is to utilize the six-axis measurement platform for orthodontic correction to precisely measure and analyze the three-dimensional force exerted on the anterior teeth when different initial torques are applied to the posterior teeth in cases involving tooth extraction. The study aims to provide valuable insights and guidance for orthodontic professionals in the clinical design of treatment plans using clear aligners for cases that require tooth extraction.

## Material and methods

**Fig. 1:**
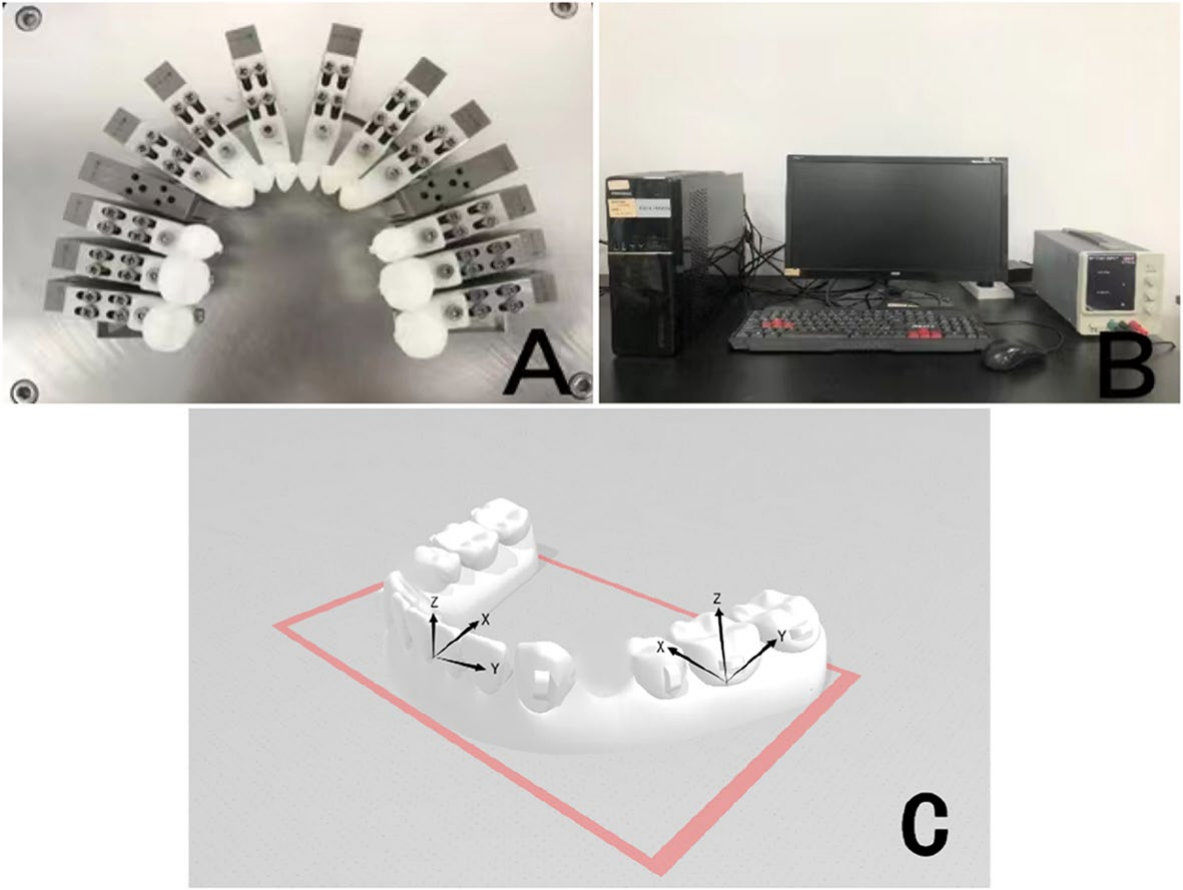
The force measurement system. **A** Three-dimensional-printed resin teeth connected separately with the multi-axis force/moment transducer by hexagonal screws. **B** The computer linked with the measurement system. **C** The coordinate system for the forces and moments measured. The x-axis represents the labiolingual/buccolingual force.The y-axis is oriented parallel to the mesiodistal direction of teeth.The z-axis runs through the center of tooth and parallel to the long axis of this tooth

The experiment utilized the Smartee 1.1 six-axis measurement platform (OrthoTech Co., Ltd., Shanghai, China) to accurately measure the three-dimensional forces and moments exerted on the target teeth. The measurement platform comprised 12 three-dimensional force/moment microsensors and 12 individual 3D printed resin mandibular teeth. Each resin tooth was affixed to a set of four fixing screws, which were then connected to their corresponding three-dimensional force/moment microsensor (Fig. 1A).

The experiment involves scanning a resin model of the mandibular standard dental arch and importing it into the Smartcheck 5.0 case design software (OrthoTech Co., Ltd., China). Four groups of mandibular dental arch models will be created, each with different initial torques applied to the posterior teeth. The groups are as follows:

- 1. Posterior teeth with Andrews standard torque.
- 2. Posterior teeth with an additional 5° buccal crown torque.
- 3. Posterior teeth with an additional 5° lingual crown torque.
- 4. Posterior teeth with an additional 10° lingual crown torque.

All four groups will be fabricated using a 3D printer (SLA rapid prototyping printer, Lite 600HD, China) and photosensitive resin. The torque and axial inclinations of all teeth, except for the second premolar, first molar, and second molar with torque variations, will remain unchanged. Horizontal rectangular attachments will be designed on the first molar and second molar, while vertical rectangular attachments will be placed on the second premolar and canine. The attachment positions will be precisely located at the clinical crown center, with dimensions of 3 mm × 2 mm × 1 mm.

A total of ninety-six sets of thermoplastic aligners will be produced using a thermoforming machine and a 0.75 mm thick thermoplastic aligner material (Crystal, OrthoTech Co., Ltd., China). The aligners will be categorized into two groups, namely Group A and Group B, based on whether they have an activation design. Within each group, further division will be made into subgroups 1 to 4, representing different initial torques applied to the posterior teeth. Each subgroup will consist of 12 sets of clear aligners. Table 1 presents the specific details regarding the grouping of aligners for Groups A and B. Group A will serve as the control group without any activation design, while Group B will be the experimental group with a design intended for overall retraction of the lower anterior teeth from 3–3.

**Table 1:** Study protocol

Research group	Amount of mandibular 3–3 inward retraction (mm)	Amount of mandibular first premolar torque(°)	Amount of mandibular first molar torque(°)	Amount of mandibular second molar torque(°)
A1	0	-22	-30	-35
A2	0	-17	-25	-30
A3	0	-27	-35	-40
A4	0	-32	-40	-45
B1	0.25	-22	-30	-35
B2	0.25	-17	-25	-30
B3	0.25	-27	-35	-40
B4	0.25	-32	-40	-45

Group A1: no activation, posterior teeth in Andrews’ standard torque group; Group A2: no activation, posterior teeth increased by 5°crown-buccal to torque group; Group A3: no activation, posterior teeth increased by 5°crown-lingual to torque group; Group A4: no activation, posterior teeth increased by 10° crown-lingual to torque group; Group B1: anterior teeth inwardly retracted by 0.25 mm, posterior teeth in Andrews' standard torque group; Group B2: anterior teeth with 0.25 mm of internal retraction and posterior teeth with 5°crown buccal to buccal torque group; Group B3: anterior teeth with 0.25 mm of internal retraction and posterior teeth with 5°crown lingual to buccal torque group; Group B4: anterior teeth with 0.25 mm of internal retraction and posterior teeth with 10°crown lingual to buccal torque group

During the sequential placement of clear aligners on the resin model of the mandibular dental arch using the orthodontic six-axis measurement platform, the computer system (Fig. 1B) captures and records approximately 40 force values within a span of 1 s. To describe the distribution of forces on the teeth in three-dimensional directions, a distinct three-dimensional coordinate system is established for each tooth (Fig. 1C).

In this system:

- The X-axis represents the labiolingual direction of the tooth, where lingual is considered positive and labial is denoted as negative.
- The Y-axis represents the mesiodistal direction of the tooth, with the positive direction running from tooth 37 to tooth 47.
- The Z-axis represents the vertical direction of the tooth, with the crown side regarded as positive and the root side as negative.

To account for calibration positioning errors, the A1-4 groups (control groups without any activation design) of clear aligners will be placed on the orthodontic six-axis measurement platform. The average force values in the three-dimensional directions for groups A1-4 will be measured and recorded (Table 2).

**Table 2:** Force distribution within clear aligner and comparisons of the forces in group A1–4

Tooth number	Force direction	A1	A2	A3	A4
37	Fx	0.29 ± 0.02	0.22 ± 0.26	0.38 ± 0.04	0.36 ± 0.67
	Fy	-0.89 ± 0.01	1.72 ± 3.15	2.18 ± 3.71	-0.56 ± 0.78
	Fz	-0.65 ± 0.06	-1.69 ± 1.93	0.11 ± 0.88	-0.57 ± 0.08
36	Fx	-2.31 ± 0.09	-2.09 ± 0.35	-1.77 ± 0.44	-2.07 ± 0.12
	Fy	-1.95 ± 0.39	-3.22 ± 1.59	-7.43 ± 1.86	-8.79 ± 0.40
	Fz	3.40 ± 0.05	3.27 ± 0.56	2.40 ± 0.25	2.56 ± 0.15
35	Fx	0.51 ± 0.07	0.65 ± 0.11	-0.53 ± 1.14	-1.39 ± 0.19
	Fy	-1.51 ± 0.37	-1.67 ± 0.32	-3.97 ± 1.21	-4.82 ± 0.29
	Fz	-8.83 ± 0.12	-9.10 ± 0.14	-9.90 ± 0.71	-10.42 ± 0.14
33	Fx	2.84 ± 0.49	3.14 ± 0.29	0.13 ± 2.78	-1.91 ± 0.62
	Fy	-5.73 ± 0.24	-5.38 ± 0.28	-5.53 ± 0.39	-5.25 ± 0.11
	Fz	6.06 ± 0.14	6.21 ± 0.11	6.55 ± 0.31	6.76 ± 0.06
32	Fx	7.98 ± 0.78	7.76 ± 0.13	5.04 ± 2.30	3.40 ± 0.54
	Fy	-5.07 ± 0.39	-4.58 ± 0.17	-4.13 ± 0.69	-3.59 ± 0.13
	Fz	19.49 ± 0.24	19.43 ± 0.08	19.23 ± 0.16	19.09 ± 0.02
31	Fx	13.10 ± 2.93	13.76 ± 0.16	10.70 ± 2.60	8.80 ± 0.61
	Fy	-3.18 ± 0.63	-2.30 ± 0.16	-2.78 ± 0.08	-2.80 ± 0.02
	Fz	17.47 ± 2.01	18.47 ± 0.12	18.99 ± 0.21	19.16 ± 0.07
41	Fx	5.52 ± 2.00	6.45 ± 1.20	3.62 ± 1.96	2.16 ± 0.42
	Fy	-3.84 ± 1.94	-6.17 ± 0.26	-3.44 ± 2.33	-1.72 ± 0.61
	Fz	13.29 ± 1.02	14.53 ± 1.48	14.54 ± 0.37	14.26 ± 0.02
42	Fx	7.70 ± 0.81	7.32 ± 0.09	4.19 ± 2.56	2.32 ± 0.59
	Fy	-5.46 ± 0.47	-5.49 ± 0.09	-3.27 ± 1.82	-1.99 ± 0.45
	Fz	13.50 ± 0.47	12.82 ± 0.15	13.20 ± 0.03	13.16 ± 0.01
43	Fx	0.53 ± 0.09	0.63 ± 0.02	0.29 ± 0.30	0.09 ± 0.08
	Fy	-3.06 ± 0.05	-3.17 ± 0.05	-3.00 ± 0.16	-2.85 ± 0.02
	Fz	12.17 ± 0.08	12.14 ± 0.06	12.01 ± 0.17	11.88 ± 0.01
45	Fx	-1.25 ± 0.08	-1.23 ± 0.06	-1.14 ± 0.12	-1.03 ± 0.04
	Fy	-4.86 ± 0.12	-4.96 ± 0.07	-4.00 ± 0.74	-3.45 ± 0.17
	Fz	-0.87 ± 0.10	-0.90 ± 0.09	-1.20 ± 0.16	-1.35 ± 0.03
46	Fx	-12.62 ± 0.06	-12.87 ± 0.26	-13.51 ± 0.50	-13.91 ± 0.15
	Fy	-4.18 ± 0.15	-4.26 ± 0.09	-2.96 ± 1.03	-2.18 ± 0.21
	Fz	4.13 ± 0.09	3.81 ± 0.40	4.40 ± 0.55	4.71 ± 0.21
47	Fx	-4.87 ± 0.05	-4.59 ± 0.17	-4.89 ± 0.23	-5.02 ± 0.07
	Fy	-2.44 ± 0.02	-2.51 ± 0.13	-1.26 ± 1.06	-0.54 ± 0.23
	Fz	-5.58 ± 0.09	-6.36 ± 1.79	-5.91 ± 0.50	-6.21 ± 0.12

Values are expressed as mean ± standard deviation. The FDI tooth numbering system was used. Group A is the reference data. Fx is the buccolingual force, and the lingual force is positive. Fy is the proximal–distal force, and it is positive in the direction from 37 distal-medial to 47 distal-medial. Fz is the vertical force, and the coronal side is positive. Fx is the buccolingual force, and the lingual force is positive

**Fig. 2:**
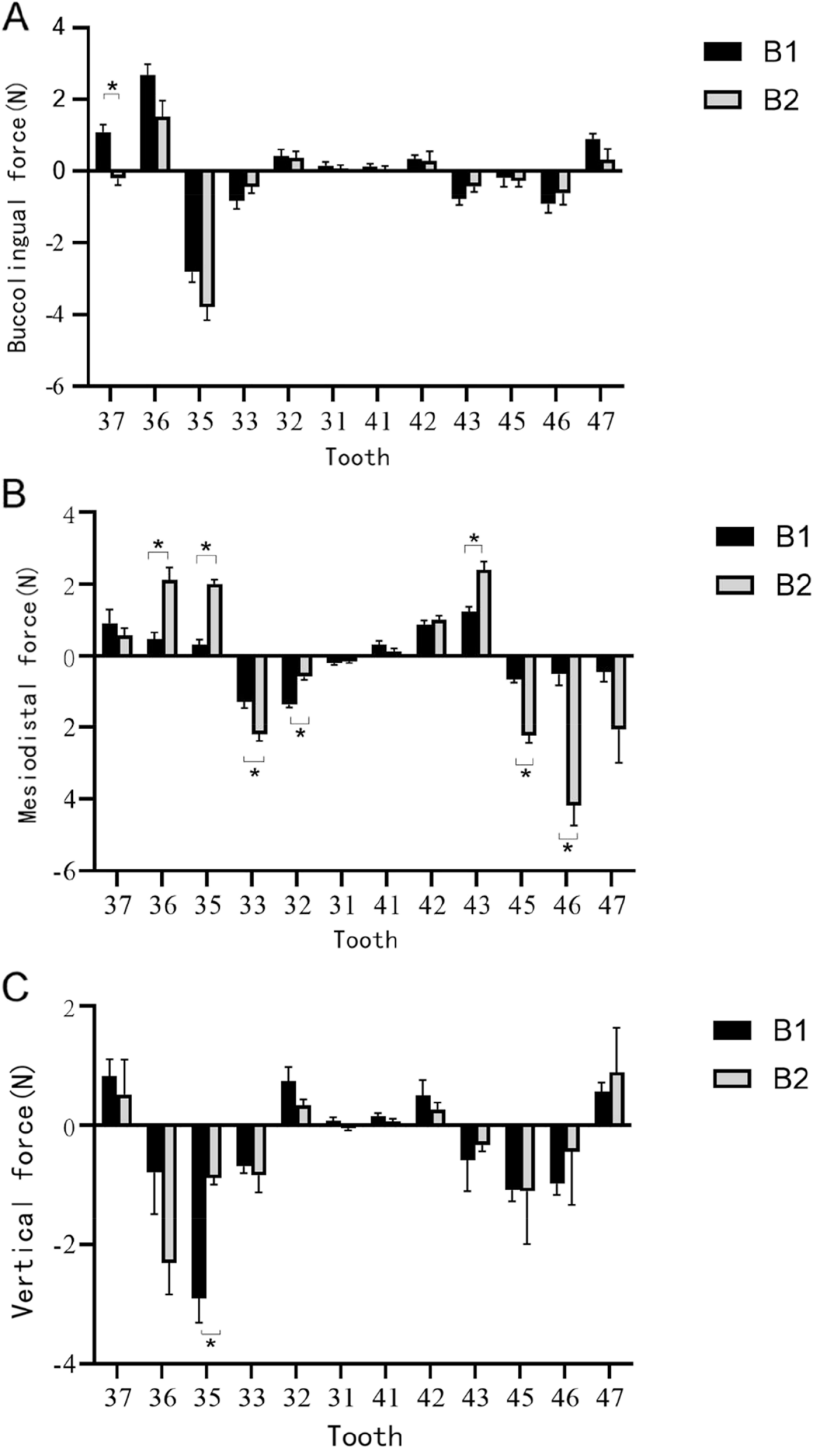
Comparisons of the three-dimensional forces in groups B1 and B2. **A** Forces in the buccolingual direction. **B** Forces in the mesiodistal direction. **C** Forces in the vertical direction. Group B1,anterior teeth inwardly retracted by 0.25 mm, posterior teeth in Andrews’ standard torque group; Group B2,anterior teeth with 0.25 mm of internal retraction and posterior teeth with 5°crown buccal to buccal torque group; *:*P* < 0.05

**Fig. 3:**
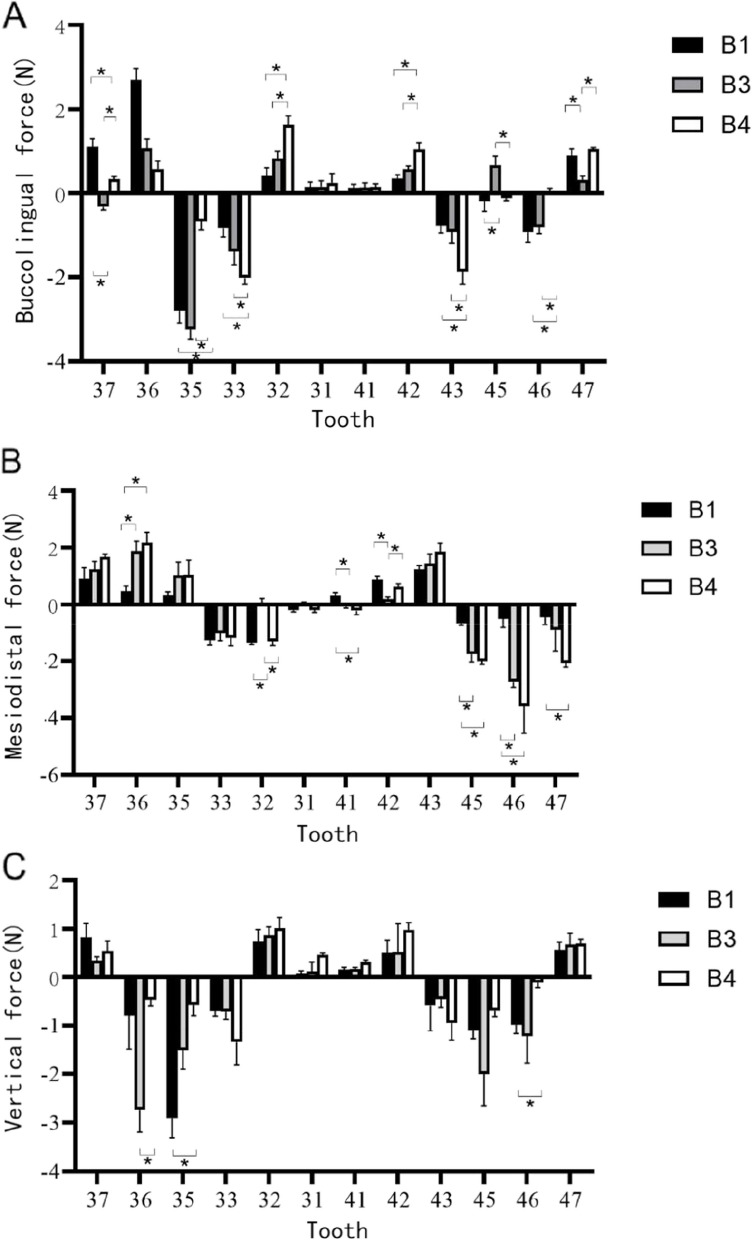
Comparisons of the three-dimensional forces in groups B1 and B2. **A** Forces in the buccolingual direction. **B** Forces in the mesiodistal direction. **C** Forces in the vertical direction. Group B1,anterior teeth inwardly retracted by 0.25 mm, posterior teeth in Andrews’ standard torque group; Group B3: anterior teeth with 0.25 mm of internal retraction and posterior teeth with 5°crown lingual to buccal torque group; Group B4: anterior teeth with 0.25 mm of internal retraction and posterior teeth with 10° crown lingual to buccal torque group.*:*P* < 0.05

Subsequently, the B1-4 groups (experimental groups with a design for overall retraction of the lower anterior teeth by 0.25 mm) of clear aligners will be sequentially placed on the orthodontic six-axis measurement platform. The forces exerted on the target teeth by each aligner will be measured and recorded. The corresponding force values measured for groups A1-4 will be subtracted from the recorded values, yielding the average force values in the three-dimensional directions for groups B1-4 (Table 3). These values will be depicted in a histogram to visualize the distribution trends of the force values (Figs. 2 and 3).

**Table 3:** Force distribution within clear aligner and comparisons of the forces in group B1–4

Tooth number	Force direction	B1	B2	B3	B4
37	Fx^#^	1.09 ± 0.69^◆▼★^	-0.20 ± 0.68^▲^	-0.33 ± 0.26^▲★^	0.33 ± 0.23^▲▼^
	Fy^#^	0.91 ± 1.32	0.57 ± 0.72^★^	1.23 ± 1.00	1.68 ± 0.32^◆^
	Fz^#^	0.82 ± 0.98	0.52 ± 1.03	0.50 ± 1.81	0.34 ± 0.29
36	Fx^#^	2.69 ± 0.98	1.52 ± 1.55	1.06 ± 0.79	0.57 ± 0.67
	Fy^&^	0.47 ± 0.62^◆▼★^	2.12 ± 1.21^▲^	1.88 ± 1.23^▲^	2.17 ± 1.27^▲^
	Fz^#^	-0.79 ± 1.42	-2.31 ± 1.81^★^	-2.74 ± 1.58^★^	-0.46 ± 0.45^◆▼^
35	Fx^#^	-2.80 ± 1.00^★^	-3.79 ± 1.26^★^	-3.24 ± 0.76^★^	-0.67 ± 0.70^▲◆▼^
	Fy^#^	0.32 ± 0.45^◆^	2.02 ± 0.39^▲^	1.03 ± 1.62	1.04 ± 1.81
	Fz^#^	-2.91 ± 1.39^◆★^	-0.88 ± 0.38^▲^	-1.51 ± 1.35	-0.57 ± 0.21^▲^
33	Fx^&^	-0.82 ± 0.76^★^	-0.44 ± 0.55^★^	-1.39 ± 1.09^★^	-2.01 ± 0.53^▲◆▼^
	Fy^&^	-1.28 ± 0.57^◆^	-2.19 ± 0.67^▲▼★^	-1.02 ± 0.92^◆^	-1.20 ± 0.95^◆^
	Fz^#^	-0.69 ± 0.39	-0.84 ± 1.00	-0.70 ± 0.56	-1.34 ± 1.62
32	Fx^&^	0.42 ± 0.63^★^	0.37 ± 0.61^▼★^	0.83 ± 0.59^◆★^	1.62 ± 0.73^▲◆▼^
	Fy^&^	-1.35 ± 0.26^◆▼^	-0.57 ± 0.36^▲▼★^	0.07 ± 0.49^▲◆★^	-1.30 ± 0.57^◆▼^
	Fz^&^	0.14 ± 0.41	0.07 ± 0.34	0.15 ± 0.49	0.25 ± 0.78
31	Fx^&^	0.14 ± 0.41	0.07 ± 0.34	0.15 ± 0.49	0.25 ± 0.78
	Fy^&^	-0.19 ± 0.22	-0.14 ± 0.19	0.04 ± 0.17	-0.19 ± 0.29
	Fz^#^	0.07 ± 0.20	-0.05 ± 0.13^★^	0.12 ± 0.82	0.46 ± 0.44^◆^
41	Fx^&^	0.13 ± 0.28	0.05 ± 0.29	0.13 ± 0.39	0.15 ± 0.26
	Fy^&^	0.31 ± 0.36^▼★^	0.11 ± 0.31^★^	-0.03 ± 0.29^▲^	-0.20 ± 0.15^▲◆^
	Fz^&^	0.16 ± 0.16	0.06 ± 0.15^★^	0.17 ± 0.12	0.31 ± 0.15^◆^
42	Fx^&^	0.34 ± 0.34^★^	0.28 ± 0.88^★^	0.57 ± 0.26^★^	1.03 ± 0.57^▲◆▼^
	Fy^&^	0.88 ± 0.36^▼^	1.01 ± 0.40^▼^	0.19 ± 0.30^▲◆★^	0.62 ± 0.40^▼^
	Fz^&^	0.50 ± 0.90	0.27 ± 0.41	0.52 ± 0.58	0.98 ± 0.85
43	Fx^&^	-0.78 ± 0.57^★^	-0.44 ± 0.48^★^	-0.92 ± 0.93^★^	-1.86 ± 1.05^▲◆▼^
	Fy^&^	1.23 ± 0.46^◆^	2.41 ± 0.78^▲^	1.43 ± 1.20	1.86 ± 1.06
	Fz^#^	-0.58 ± 0.82	-0.33 ± 0.37	-0.45 ± 0.59	-0.94 ± 1.25
45	Fx^&^	-0.19 ± 0.85^▼^	-0.28 ± 0.56^◆^	0.67 ± 0.73^▲◆★^	-0.12 ± 0.21^▼^
	Fy^#^	-0.66 ± 0.30^◆▼★^	-2.23 ± 0.76^▲^	-1.76 ± 0.95^▲^	-2.00 ± 0.41^▲^
	Fz^#^	-1.08 ± 0.64	-1.11 ± 1.08	-2.00 ± 2.27	-0.68 ± 0.43
46	Fx^#^	-0.92 ± 0.85^★^	-0.62 ± 1.10	-0.81 ± 0.52^★^	0.04 ± 0.28^▲▼^
	Fy^#^	-0.50 ± 1.10^◆▼★^	-4.17 ± 0.96^▲^	-2.73 ± 0.70^▲^	-3.58 ± 3.29^▲^
	Fz^#^	-0.98 ± 0.66^★^	-0.44 ± 1.10	-1.22 ± 5.41	-0.11 ± 0.34^▲^
47	Fx^#^	0.90 ± 0.53^▼^	0.32 ± 1.03	0.31 ± 0.31^▲★^	1.05 ± 0.11^▼^
	Fy^#^	-0.45 ± 0.94^★^	-2.07 ± 1.17	-0.91 ± 2.56	-2.07 ± 0.54^▲^
	Fz^#^	-0.56 ± 0.55	0.89 ± 1.59	0.67 ± 0.78	0.69 ± 0.31

Values are expressed as mean ± standard deviation. The FDI tooth numbering system was used. Values in group B were obtained by subtracting them from the corresponding values in group A. Fx is the buccolingual force, which is positive. fy is the proximal–distal-medial force, which is positive in the direction of 37 distal-medial to 47 distal-medial. fz is the vertical force, which is positive in the coronal direction.

& represents the use of the Bonferroni test; # represents the use of Dunnett’s T3 test. Comparisons between groups, *P* < 0.05, with differences labeled ▲ with group A1, ◆ with group A2, ▼ with group A3, and ★ with group A4

To minimize human errors during the placement of clear aligners, a consistent and uniform approach will be followed by the same operator, employing the same method and force for each set.

The gathered data will undergo statistical analysis using SPSS 26.0. For continuous data that follow a normal distribution, the results will be presented as “mean ± standard deviation”. One-way analysis of variance (ANOVA) will be conducted to examine the differences in three-dimensional force among the various groups of anterior teeth. In the case of meeting the assumption of homogeneity of variances, Bonferroni post-hoc tests will be utilized. However, if the assumption is violated, Dunnett's T3 test, which accounts for unequal variances, will be employed. The significance level will be set at α = 0.05, where a *P*-value below 0.05 will indicate statistical significance for observed differences.

## Results

Since the A1-4 groups (control groups without any activation design) serve solely for calibration and do not have any specific force application, the data for Group A will not be analyzed further in the subsequent discussion. The focus will be on the data from the experimental groups (B1-4) with a design for overall retraction of the lower anterior teeth.

In the B1-4 groups of dental arches (experimental groups with a design for overall retraction of the lower anterior teeth by 0.25 mm), the forces and trends in the buccolingual and mesiodistal directions are generally consistent within the mandibular central incisor group (2–2) and the mandibular canine group (bilateral canines). Therefore, in the subsequent discussion, we will collectively refer to the mandibular central incisor group and bilateral canine group as the incisor group and canine group, respectively.

In the mandibular posterior teeth with standard initial torque, the forces exerted on the teeth can be described as follows:

- 1. Buccolingual direction: The incisor group experiences mild to moderate lingual forces, approximately 0.77N. Conversely, the canine group experiences larger labial forces, approximately 0.80N.
- 2. Mesiodistal direction: Both the incisor group and the canine group primarily experience distal forces, approximately 0.87N.
- 3. Vertical direction: The incisor group experiences mild elongation forces, approximately 0.21N. And, the canine group experiences mild to moderate intrusion forces, approximately 0.63N.

When comparing the mandibular posterior teeth with initial labial torque to those with standard initial torque, the forces exerted on the teeth can be described as follows:

- 1. Buccolingual direction: The incisor group in the labial torque group experiences slightly reduced lingual forces compared to the standard torque group by approximately 0.57N. However, the difference is not statistically significant (*P* > 0.05). Similarly, the canine group in the labial torque group also experiences a decrease in labial forces by approximately 0.36N, but the difference is not statistically significant (*P* > 0.05).
- 2. Mesiodistal direction: In the labial torque group, the incisor group experiences a decrease in distal forces by approximately 0.41N compared to the standard torque group. The difference between the two groups is statistically significant for the left incisor group (*P* < 0.05). On the other hand, the canine group in the labial torque group experiences an increase in distal forces by approximately 1.44N compared to the standard torque group, and the difference between the two groups is statistically significant for the canine group (*P* < 0.05).
- 3. Vertical direction: The incisor group in the labial torque group experiences a slight decrease in elongation forces by approximately 0.12N compared to the standard torque group. However, the difference is not statistically significant (*P* > 0.05). For the canines, the left canine experiences an increase in elongation forces by 0.15N in the labial torque group, while the right canine experiences a decrease in elongation forces by 0.25N. However, these differences are not statistically significant (*P* > 0.05).

When comparing the mandibular posterior teeth with standard initial torque to those with initial 5° and 10° lingual torque, the forces exerted on the teeth can be described as follows:

- 1. Buccolingual direction: Both the incisor groups at initial lingual inclinations of 5° and 10° exhibit greater lingual forces compared to the initial standard torque group of the mandibular posterior teeth. There were statistically significant differences in bilateral lateral incisors between the initial torque group of the mandibular posterior teeth at 10° lingual inclination and the initial standard torque group, as well as between the initial torque group of the mandibular posterior teeth at 10° lingual inclination and the initial torque group of the mandibular posterior teeth at 5° lingual inclination (all *P* < 0.05). Moreover, the force exerted on the mandibular posterior teeth in the initial 10° lingual torque group is approximately 0.33N greater than that in the initial 5° lingual torque group,And the cuspid groups all experience greater labial forces. Similarly, there were statistically significant differences between the initial torque group of the mandibular posterior teeth at 10° lingual inclination and the initial standard torque group, as well as between the initial torque group of the mandibular posterior teeth at 10° lingual inclination and the initial torque group of the mandibular posterior teeth at 5° lingual inclination (all *P* < 0.05). Moreover, the force exerted on the mandibular posterior teeth in the initial 10° lingual torque group is approximately 0.78N greater than that in the initial 5° lingual torque group.
- 2. Mesiodistal direction: The incisor group primarily experiences smaller distal forces in both the 5° and 10° lingual torque groups compared to the standard torque group. The differences between the 5° lingual torque group and the standard torque group, as well as the 10° lingual torque group, are statistically significant for both the bilateral incisor group and the bilateral canine group (*P* < 0.05). Moreover, the force exerted on the mandibular posterior teeth in the initial 10° lingual torque group is approximately 0.45N greater than that in the initial 5° lingual torque group. Specifically, there is a statistically significant difference between the standard torque group and the 5° and 10° lingual torque groups in the right central incisor (*P* < 0.05). The mandibular canine group experiences distal forces in all three groups, but there are no statistically significant differences between the groups for both the bilateral canine group (*P* > 0.05).
- 3. Vertical direction: The incisor group experiences larger elongation forces in both the 5° and 10° lingual torque groups compared to the standard torque group.The torque groups at 5° and 10° lingual inclination are approximately 0.16N greater than the initial standard torque group, but there is no statistically significant difference between the three groups (*P* > 0.05).The mandibular canine group experiences mild to moderate intrusion forces, and there are no statistically significant differences between the groups for both the bilateral canine group (*P* > 0.05).

## Discussion

In the process of closing extraction spaces during orthodontic treatment with clear aligners, distinct movement patterns emerge in the upper and lower dental arches. Research indicates that the mandible has a higher density [18, 19] and the lower dental arch is more responsive to elastic forces [20]. Furthermore, studies reveal that individuals with untreated crowding in the lower posterior region frequently exhibit buccolingual inclination of their molars [21]. Nevertheless, it remains uncertain whether varying initial buccolingual positions of the lower posterior teeth affect the distribution of forces in the lower anterior teeth while wearing clear aligners.

In this experiment, we focus on the lower dental arch as the target arch and employ the orthodontic six-axis measurement platform to investigate how varying initial torques in the lower posterior teeth impact the three-dimensional force distribution in the lower anterior teeth throughout orthodontic treatment with clear aligners.

In the B1 group, which was designed for a 0.25 mm retraction of the mandibular anterior teeth, distinct force patterns are observed in various teeth. The mandibular incisors encounter noticeable lingual and elongation forces, while the canines experience greater labial forces and intrusion forces. The second premolars primarily undergo intrusion forces, and the second molars experience mesial and elongation forces. This phenomenon bears similarity to the clinical “roller effect” and is in line with the findings of Zhu et al.’s study [22]. In this scenario, the second molars undergo elongation, while the canines and second premolars are intruded by clear aligners, resulting in an increased Spee curve and a tilting motion of the anterior and posterior teeth toward the extraction spaces.

This phenomenon may be attributed to the fact that a 0.25 mm overall shortening was employed in this study to achieve anterior tooth retraction with clear aligners. Since the retraction force doesn’t align with the mandibular incisors’ center of resistance, the incisors experience lingual forces. The canines, positioned at the dental arch’s corner, undergo increased horizontal spacing during overall retraction of the mandibular anterior teeth, causing labial forces to act upon them. As a cohesive unit, the posterior teeth encounter mesial forces.

In the B2 group, we introduced an additional 5° buccal torque to the mandibular posterior teeth to assess its mechanical impact on the mandibular anterior teeth when the posterior teeth are upright. In comparison to the B1 group, we observed that when the mandibular posterior teeth are buccally inclined, the incisor group experiences reduced mesial and lingual forces. This can be attributed to the fact that the initial 5° buccal torque in the mandibular posterior teeth results in a more upright positioning within the dental arch. Consequently, there is an improved alignment angle between the anterior and posterior segments of the dental arch when wearing clear aligners, reducing deformations caused by their usage. This, in turn, leads to a tighter fit between the clear aligners and the mandibular resin teeth, minimizing additional lingual and mesial forces acting on the mandibular incisors due to misalignment between the aligners and the teeth.

These findings suggest that in clinical treatment, when the initial torque for the posterior teeth is buccal inclination, only a small amount of positive torque design for the incisor group is necessary for achieving overall anterior tooth retraction.

Within the B2 group, the canines exhibit a decrease in labial forces, mirroring the reduction in lingual forces observed in the incisor group. However, the canines experience an increase in mesial forces. This change can be attributed to the more upright positioning of the mandibular posterior teeth, leading to an amplification of forces in the mesial-distal direction when wearing clear aligners. Consequently, mesial forces on the mandibular posterior teeth increase, and following the interaction of forces, the mesial forces on the canines also rise. Through clinical research, Cong et al. [23] found that clear aligners exhibit a higher expression rate of mesial force on mandibular posterior teeth and have better control over buccal torque than lingual torque, which is consistent with the results of this experiment.

These findings indicate that when a patient's initial torque for the posterior teeth involves buccal inclination, it’s advisable to incorporate an adjustment in the design to introduce greater mesial forces on the canines during overall anterior tooth retraction. This adjustment can help prevent tilting movements of the canines in the mesial-distal direction.

When the initial torque of the mandibular posterior teeth is lingually inclined, the forces affecting the mandibular anterior teeth are as follows:

Clinical studies and practical experience have demonstrated that patients with malocclusion frequently present with lingual inclination of the mandibular posterior teeth [21]. Consequently, in this experiment, we compared groups B3 and B4, in which 5° and 10° lingual torque was applied to the posterior teeth, with the mandibular posterior teeth initially set at the standard torque. This comparison aimed to examine the impact of different initial lingual torques on the forces influencing the mandibular anterior teeth.

In groups B3 and B4, we observed that the forces acting on the incisor group closely resembled those in group B1. They all experienced lingual and elongation forces. Notably, as the initial lingual torque of the posterior teeth increased, the incisor group encountered augmented lingual and elongation forces. This effect can be attributed to the heightened lingual inclination angle of the mandibular posterior teeth, which reduces the common alignment angle between the anterior and posterior segments of the dental arch. To facilitate aligner placement, it becomes necessary to twist the aligner lingually on both sides of the posterior teeth, leading to aligner deformation. This deformation diminishes the fit between the anterior segment of the aligner and the mandibular resin teeth, resulting in additional lingual and elongation forces on the incisors. Furthermore, as the lingual torque of the mandibular posterior teeth intensifies, aligner deformation during placement increases, leading to greater forces on the incisors. This phenomenon bears similarity to the clinical “bowing effect” [24], where lingual tilting of the incisors is often accompanied by an increase in their vertical dimension.

Hence, when the posterior teeth exhibit an initial lingual torque inclination, it’s crucial to design a comprehensive retraction of the mandibular anterior teeth from 3 to 3. Nevertheless, it’s important to recognize that maintaining control over the torque and vertical dimension of the incisors during retraction can be challenging. Therefore, during the retraction process, it becomes necessary to enhance the labial torque design on the incisors and implement appropriate extrusion to prevent excessive overbite of the anterior teeth.

In groups B3 and B4, the canine group encounters forces similar to those in group B1, specifically labial and extrusive forces. Furthermore, as the initial lingual torque of the mandibular posterior teeth intensifies, the canines also experience heightened labial forces. This pattern parallels the increased lingual forces observed in the incisor group of groups B3 and B4, but in the opposite direction.

Regarding this phenomenon, studies [25, 26] have indicated that when orthodontic forces are applied to target teeth, adjacent teeth experience significant reactionary forces. While the measured values of these reactionary forces are only half of the applied forces on the target teeth, their clinical implications remain substantial. This suggests that when the mandibular posterior teeth exhibit an initial lingual torque inclination, conducting comprehensive mandibular anterior retraction from 3 to 3 may lead to labial movement of the mandibular canines. Moreover, as the initial lingual torque of the mandibular posterior teeth intensifies, the labial forces acting on the canines also increase. This scenario is more likely to result in cortical bone resistance during the canine movement, diminishing its efficiency and impeding the closure of the extraction space.

### Limitations

This study offers valuable mechanical insights for clinicians regarding the three-dimensional control of anterior teeth in patients undergoing orthodontic treatment with extractions while considering the various initial torques of the posterior teeth. However, it does come with certain limitations.

Firstly, this study is restricted to the mandibular dental arch. Liu et al. [20] have suggested that different biomechanical effects may occur between the maxillary and mandibular dental arches when using clear aligners for patients with extractions, owing to their distinct morphologies. Consequently, the force trends on the maxillary anterior teeth might differ from those on the mandibular anterior teeth.Secondly, due to the absence of simulations for the periodontal ligament and alveolar bone, the results obtained from this study cannot entirely predict tooth movement outcomes in clinical practice.Lastly, this experiment is an in vitro study, and the presence of saliva between the clear aligners and the dentition in the clinical setting introduces friction, which was not accounted for in this study.

Despite these limitations, this study marks an initial exploration of the effects of various initial torques on the mandibular posterior teeth concerning the three-dimensional forces acting on the mandibular anterior teeth during comprehensive anterior retraction with clear aligners following extractions. It provides a reference for designing clear aligners to control tooth movement, although further research will be essential in the future.

## Conlusions

- 1. In the context of orthodontic treatment with clear aligners following extractions, varying initial torques of the mandibular posterior teeth exert significant influence on both buccolingual and anteroposterior forces applied to the mandibular anterior teeth.
- 2. When the mandibular posterior teeth exhibit an initial buccal torque, it is advisable to reduce the positive torque design for the incisors and augment the design for the canines in the anteroposterior direction.
- 3. In cases where the mandibular posterior teeth have an initial lingual torque, it is recommended to increase the positive torque design for the incisors and incorporate additional lingual design for the mandibular canines, particularly as the lingual inclination angle intensifies. This precautionary measure helps mitigate excessive lip movement of the mandibular canines and the potential development of a deep overbite during tooth movement, which may encounter cortical bone resistance.

### Supplementary Information

**Supplementary Material 1. **

## Data Availability

The datasets used and/or analyzed during the current study are available from the corresponding author on reasonable request.
